# Ectopic thyroid tissue in the head and neck: a case series

**DOI:** 10.1186/1756-0500-7-790

**Published:** 2014-11-06

**Authors:** Croce Adelchi, Pugliese Mara, Laus Melissa, Alessandro De Stefano, Mantini Cesare

**Affiliations:** Department of Medical Oral Sciences, and Biotechnology, Section of Otolaryngology, “G. D’Annunzio” University, Chieti - Pescara, Italy; Department of Otorhinolaryngology, University of Chieti, Via dei Vestini 5, Chieti, 66013 Italy; Department of Neuroscience and Imaging, Section of Diagnostic Imaging and Therapy – Radiology Division, “G. d’Annunzio” University, Chieti - Pescara, Italy

**Keywords:** Thyroid gland, Ectopic thyroid tissue, Surgery

## Abstract

**Background:**

Through a review of three cases, the etiopathogenetic, clinical-diagnostic, and therapeutic aspects of ectopic thyroid tissue are herein discussed to highlight the main presentations of this polymorphous disease.

**Case presentations:**

The first case involved an ectopic thyroid gland in the lingual area in a 45-year-old Caucasian woman who presented with dysphagia and midline swelling at the base of the tongue. The second case involved a 22-year-old Caucasian woman with a submandibular mass comprising ectopic thyroid tissue. The third case involved a 33-year-old Caucasian man with a typical thyroglossal duct cyst characterized by the presence of thyroid tissue upon histological analysis.

**Conclusion:**

Surgery seems to be the most appropriate treatment for patients with ectopic thyroid tissue showing clinical signs of upper airway obstruction or when the lesion shows signs of infection or malignant degeneration. When a site of ectopic thyroid tissue is the only such site in the body, removal of this tissue will usually lead to hypothyroidism that requires medical thyroid hormone replacement.

## Background

There are several circumstances in which normal or abnormal thyroid tissue may be found within the neck but outside the thyroid gland. Rosai and Ackerman’s classification [1] distinguishes such tissue as follows:

1. Ectopic thyroid tissue resulting from faulty embryogenesis.
2. Hyperplastic thyroid tissue outside the gland in patients with Graves’ disease.
3. Mechanical implantation of thyroid tissue in the neck secondary to surgical intervention or accidental trauma.
4. A sequestered thyroid nodule, also known as a parasitic or accessory nodule; i.e., the occurrence of a peripherally located thyroid nodule in which the anatomic connection with the main gland is either lost or missed by the surgeon.
5. Thyroid tissue within cervical lymph nodes, which may develop by two unrelated processes: metastases of clinically undetected thyroid carcinomas, nearly always of the papillary variety (most cases), or the development of normal follicles within lymph nodes.
6. Thyroid tissue as a component of a teratoma, particularly in the ovary.

The development of ectopic thyroid tissue secondary to faulty embryogenesis is one of the most frequently encountered clinical situations involving thyroid tissue abnormalities. In the last 10 years, we have observed three such cases, which are herein described. Passage of the thyroid gland through the neck is a very critical and delicate step during embryonic development. In fact, the anlage of the thyroid appears within the embryo as a midline structure at the site corresponding to the foramen cecum of the adult tongue. From here, it descends as a component of the thyroglossal duct along the midline to reach its final position in the mid-neck. The thyroglossal duct is usually situated anterior to the hyoid bone, which divides it into a suprahyoid and infrahyoid portion. In the normal course of events, the thyroglossal duct is obliterated and disappears while the anlage of the thyroid simultaneously expands laterally to form the thyroid lobes [1, 2]. Thyroglossal duct anomalies are caused by localized persistence of the thyroglossal duct and may be accompanied by a cystic dilatation secondary to secretion from the lining cells. The ectopic thyroid tissue is commonly found in the walls of thyroglossal duct cysts; it appears in the form of small groups of follicles and is present in 25% to 65% of histologically examined cysts. The frequency of ectopic thyroid tissue is related to the number of sections submitted for histologic examination.

**Figure 1:**
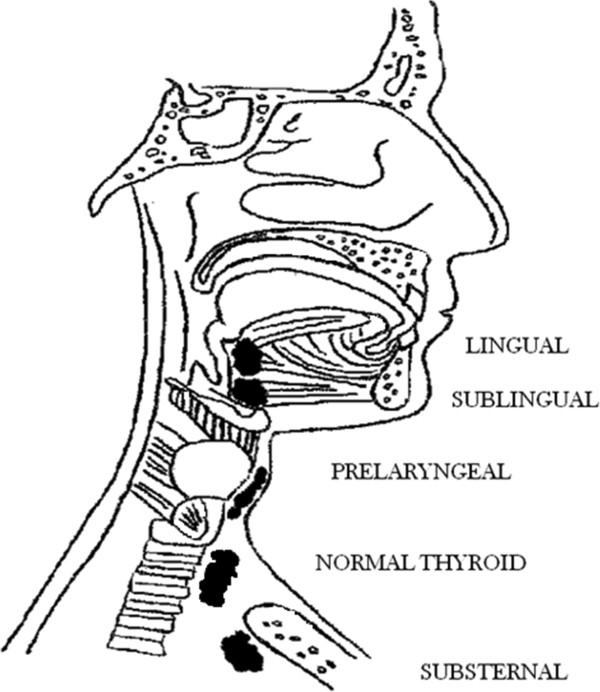
**Distribution of heterotopic thyroid tissue.**

Ectopic thyroid tissue can be found not only as a component of thyroglossal duct cysts, but anywhere along the course of the thyroglossal duct [1, 3, 4]. The most frequent location is the base of the tongue, where the presence of ectopic thyroid tissue may result in swallowing difficulty and respiratory obstruction. Other sites include the anterior tongue, submandibular or sublingual region, larynx, trachea, mediastinum, and heart [5, 6] (Figure 1). This ectopic thyroid tissue does not differ microscopically from that seen in the main thyroid gland [7].

## Case presentations

### Case 1

A 45-year-old Caucasian woman presented with a history of swelling at the base of the tongue, first noticed about 20 years before hospitalization. The swelling gradually increased in size and eventually caused dysphagia, inspiratory dyspnea, and stomatolalia. The patient’s birth history, developmental milestones, and menstrual history were normal. She was a smoker (10 cigarettes/day). Her family history revealed that one sister was affected with thyroid goiter and had undergone surgery. No hypothyroidism symptoms were referred.

On clinical examination, a 3 × 4-cm round mass was noted at the base of the tongue. The mass occupied the entire oropharyngeal space with the exception of a small portion. Open surgical biopsy revealed thyroid tissue with focal cystic dilatation of follicular cells. Ultrasonography showed the absence of the thyroid gland at its usual site.

The patient was successfully treated with local excision of the lingual thyroid tissue through a median vertical suprahyoid approach and tracheotomy at the second to third tracheal rings. The hyoid bone was incised along the middle, the prelaryngeal muscles were parted, and a median pharyngotomy was created through the thyrohyoid space. The mass was then removed. Drainage tubes were placed at the base of the tongue and in the suture lodge.

Following surgery, repeat ultrasonography of the neck revealed no abscesses or problems at the site of intervention. Histological examination confirmed removal of lingual thyroid tissue containing a small node of parathyroid tissue. Removal of the heterotopic thyroid tissue required subsequent medical replacement therapy to maintain thyroid function.

### Case 2

**Figure 2:**
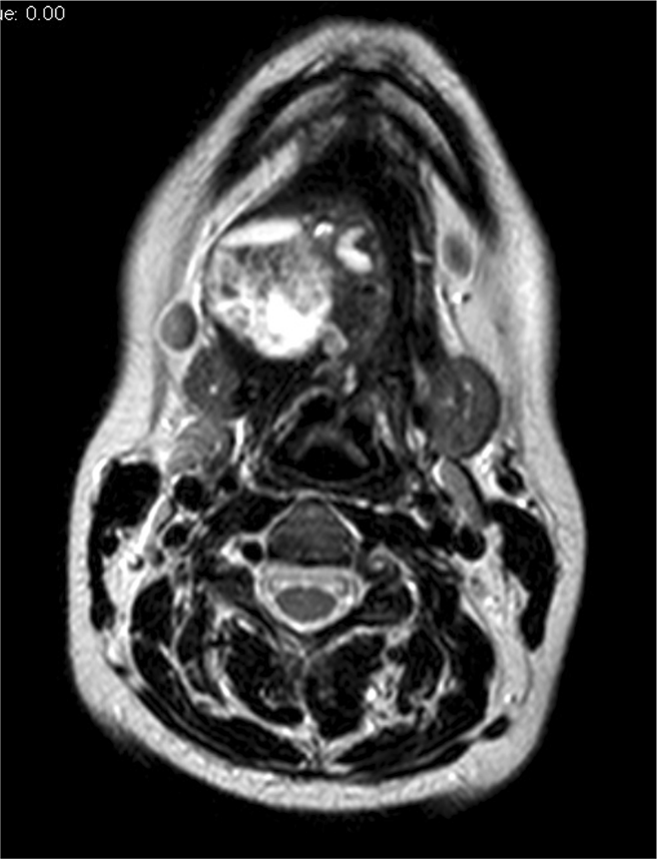
**Axial T2-weighted turbo spin echo unenhanced sequence without fat saturation shows a 4.5 × 4.0 × 3.7-cm hyperintense, rough oval formation suspected to contain mucus.**

A 22-year-old Caucasian woman presented for evaluation of a right submandibular swelling exhibiting slow onset (about 3 years). She reported a history of chronic thyroiditis with hypothyroidism treated with thyroxin 50 mcg/day.Neck ultrasonography showed a 4 × 5-cm nodule of mostly solid composite structures located near the right submandibular gland. The thyroid gland was normal in morphology and size, but exhibited features consistent with chronic thyroiditis (hypoechogenic structure, soft edges). Magnetic resonance imaging showed a 4.5 × 4.0 × 3.7-cm, rough oval right submandibular lesion at the level of the floor of the mouth; the lesion was suspected to contain mucous (Figure 2). The lesion was displacing the intrinsic muscles of the tongue and the submandibular gland. Multiple variably sized lymph nodes with a reactive appearance were present in the lateral neck, jaw, and chin regions.

After evaluating her examination findings, the patient underwent surgery. We isolated the normal submandibular gland through a right submandibular skin incision. The gland was not connected to the nodular formation. Its dimensions were 4 × 4 cm, and it was encapsulated and exhibited a hard elastic consistency. Immediately after removal of the tissue, extemporaneous histological examination revealed a diagnosis of ectopic thyroid tissue. The surgical intervention was concluded with application of intradermal sutures and a dressing.

Postoperative thyroid function tests revealed a thyroid-stimulating hormone (TSH) level of 4226 IU/ml (reference range, 0.250–4.500 IU/ml), free thyroxine level of 1.21 ng/dL (reference range, 0.70–1.70 ng/dL), and free triiodothyronine level of 1.69 pg/mL (reference range, 2.00–4.90 pg/mL). The endocrinologist prescribed thyroxin at 50 mcg/day upon discharge. The definitive histological diagnosis, which came to our attention after the patient had been discharged, was hypertrophic thyroid tissue in an adenomatous-type stroma.

### Case 3

**Figure 3:**
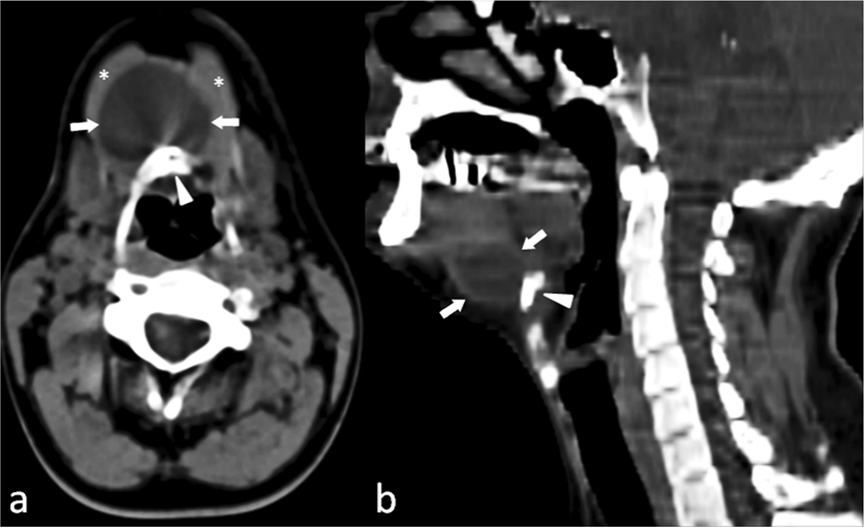
**Axial (a) and sagittal (b) unenhanced computed tomography scans show the presence of a hypodense round mass (3 × 4 cm) (arrows) with a fluid density value (mean density = 20 HU) located medially and in front of the hyoid bone (arrowhead).**

A 33-year-old Caucasian man presented with a midline fistula originating from a neck swelling compatible with a midline cyst of the neck (thyroglossal duct cyst). The cyst became periodically infected during the 5 years before hospitalization. The patient’s birth history and developmental milestones were normal. He was a smoker (20 cigarettes/day) and a former heroin addict. The patient was negative for hepatitis C virus, hepatitis B virus, and human immunodeficiency virus. His family history revealed no other family members with thyroid disease.On clinical examination, a hard sclerotic area was discernible around a small (1.0 × 1.5 cm), retracted dermal scar above the hyoid bone. The patient underwent unenhanced computed tomography of the neck, which revealed a hypodense, superficial lesion of 3 cm length and many reactive lymph nodes along the jugular chain bilaterally (Figure 3). The lesion extended caudally, passing through the thyroid cartilage and laterally displacing the mylohyoid muscles.

The patient was successfully treated with local excision of a midlinemedium cyst of the neck with a hyoid bone splinter and an anterior cervical lozenge skin. Drainage tubes were placed in the suture lodge, and antibiotic therapy was begun. Histologic examination identified intracystic thyroid tissue without atypia.

## Discussion

Embryonic development of the thyroid with median endodermal thickening about 24 days after fertilization in the floor of the primitive hypopharynx; the primitive gland then descends toward its natural site, which is closely related to the hyoid bone and laryngeal cartilage [8, 9]. The thyroglossal duct is a narrow tube connecting the developing thyroid gland to the tongue, and it usually involutes at the sixth or eighth week. The foramen cecum is the opening of the thyroglossal duct in the tongue.

Descent of the thyroid during embryogenesis may not proceed normally. Thyroid descent may stop at various sites from the base of the tongue to any site of the thyroglossal duct [10, 11], resulting in the development of ectopic thyroid. Ectopic thyroid is an embryological aberration defined as “thyroid tissue not located anterolaterally to the second and fourth tracheal cartilages” [9]. Common sites of ectopic thyroid tissue in the head and neck include the base of the tongue, submandibular or sublingual sites (between the geniohyoid and mylohyoid muscles), and prelaryngeal sites (in front of the larynx and above the hyoid bone). Rare sites include the pharynx, esophagus, trachea, and, among the extracervical sites, the mediastinum, precardial sac, heart, breast, lung, duodenum, small intestinal mesentery, and adrenal gland [9, 12–17].

The cause of ectopic thyroid tissue remains unclear in most cases. Mutation of thyroid transcription factor 2, which is required for the downward migration of the thyroid gland, has been proposed as a possible mechanism [18, 19]. Additionally, the incidence of thyroid ectopy is unknown. Postmortem studies have suggested that asymptomatic thyroid tissue may be found along the path of the thyroglossal duct in as many as 7% to 10% of adults [20]. Ectopic thyroid tissue may coexist with eutopic thyroid [7, 21, 22] or may be the only functioning tissue [23–25].

Ectopic thyroid occurs more frequently in female patients, with a female:male ratio of 4:1. It is seen at any age, but occurs more commonly during childhood during adolescence, and around menopause. This probably occurs because the demand for thyroid hormones increases during these stages, increasing the circulating TSH levels with growth of ectopic thyroid tissue [14, 26]. According to several previous studies, about 33% to 62% of all patients with ectopic thyroid tissue develop hypothyroidism with increased TSH levels [9, 13, 27]. Most ectopic thyroid tissue is asymptomatic and does not require therapy.

Symptoms of ectopic thyroid tissue are related to the growth of the thyroid tissue, causing dysphagia, dysphonia with stomatolalia, bleeding, or dyspnea [9, 28–30].

All diseases capable of affecting the normal thyroid can affect the ectopic thyroid, such as adenoma, hyperplasia, inflammation, and malignancy [31–33]. The rate of malignant transformation in ectopic thyroid tissue is not greater than that in the normally positioned thyroid.

The most important diagnostic modality for ectopic thyroid is thyroid scanning with technetium-99 m. However, fine-needle aspiration cytology (which can be difficult in some sites), ultrasonography, computed tomography, and magnetic resonance imaging may help to define the extension and location of the ectopic thyroid gland. Thyroid scanning also detects the presence of other sites of thyroid tissue [5, 34].

Differential diagnoses of ectopic thyroid tissue include lymphangioma, minor salivary gland tumors, midline branchial cysts, thyroglossal duct cysts without thyroid tissue, epidermal and sebaceous cysts, hemangioma, adenoma, fibroma, and lipoma [13, 35].

Although asymptomatic and euthyroid patients do not require any treatment, they should be followed up and evaluated for any complications. Patients with high TSH levels with swelling should undergo replacement therapy with thyroid hormone, which can produce a slow reduction in the size of the mass [5, 34]. When medical treatment fails or evidence of obstructive symptoms, hemorrhage, or suspicion of malignancy is present, then surgery should be considered [5, 34].

The three herein-described cases observed in the last 10 years are interesting because each has its own features regarding the possible localization and clinical pattern of ectopic thyroid tissue. The tongue is the most frequent ectopic location of the thyroid gland [36]. Lingual thyroid tissue develops in 1 of 100,000 individuals; however, the true incidence is unknown because many patients are asymptomatic until later in life or do not present for medical treatment [14, 37, 38]. The diagnosis is usually made as a result of the incidental discovery of a mass on the back of the tongue in an asymptomatic patient. The mass may enlarge and cause dysphagia, dysphonia, dyspnea, or a sensation of choking [39]. Hypothyroidism is often present and may cause the mass to enlarge and become symptomatic; however, hyperthyroidism is very unusual [19].

Case 1 involved a 45-year-old Caucasian woman with a history of lingual thyroid that caused dysphagia, dysphonia, dyspnea, and a sensation of choking. The lingual thyroid tissue was located at the base of the tongue and occupied the entire oropharyngeal space with the exception of a small portion. For these reasons, the patient underwent surgical treatment and subsequent medical thyroid hormone replacement therapy. Interestingly, a small node of parathyroid tissue was found within the surgical specimen. This represents the most common clinical situation reported in the literature because the persistence of the thyroglossal duct is more frequent while the presence of thyroid tissue within the cyst is very rare.

In Case 2, sublingual ectopic thyroid tissue was found at a midline position above the hyoid bone. Hypothyroidism is commonly present in such patients because of the absence or hypofunction of the normal thyroid gland in most instances. An enlarging mass commonly develops during infancy, childhood, or later life. Such masses are often mistaken for thyroglossal duct cysts because they are usually located in the same anatomic position [19, 40].

Sublingual ectopic thyroid generally occurs along the midline; its presence lateral to the midline is rare. In the present study, we observed a patient with ectopic thyroid tissue located in the right submandibular gland; the thyroid gland was in its normal site, but was hypofunctional. An ectopic thyroid gland in the submandibular gland and intratracheal regions is also very rare [16, 41]. The patient presented to us due to a submandibular swelling. Her medical history included treatment for chronic thyroiditis with hypothyroidism. Surgical intervention was performed according to the patient’s test results and clinical history, and good outcome was obtained.

Case 3 involved a patient with a history of a thyroglossal duct cyst complicated by a fistula. Incomplete atrophy of the thyroglossal tract or retained epithelial cysts provide the basis for thyroglossal duct cyst development. These cysts are the most common anomalies of thyroid development seen in clinical practice [42]. Most appear in early childhood, while the rest become apparent only after the age of 30 years [43]. A thyroglossal remnant may take the form of a cyst, tract or duct, fistula, or ectopic thyroid tissue within a cyst or duct. In the present case, recurrent infection necessitated surgery. The peculiarity of this case lies in the rarity of the thyroid tissue within the intracystic specimen.

## Conclusions

Lingual, sublingual, and submandibular with prelaryngeal ectopic thyroid tissue are rare developmental anomalies of the thyroid gland. Evaluation of the differential diagnoses, including lymphangioma, minor salivary gland tumors, midline branchial cysts, thyroglossal cyst duct (without thyroid tissue), epidermal and sebaceous cysts, angioma, adenoma, fibroma, and lipoma, is an important step in patient evaluation. Treatment may be conservative with substitutive hormone treatment in patients with mild symptoms, while surgery seems to be the most appropriate treatment for patients showing clinical signs of upper airway obstruction or with a lesion showing signs of infection or malignant degeneration. In spite of its rarity, ectopic thyroid tissue is a pathological condition that must always be kept in mind in patients with oropharyngeal and neck swelling.

## Consent

Written informed consent was obtained from all three patients for publication of this Case Series and any accompanying images. A copy of the written consent is available for review by the Editorin-Chief of this journal.

## Abbreviations

MRI: Magnetic risonance imaging
TSH: Thyroid stimulating hormone
fT3: Free triiodothyronine
fT4: Free thyroxine
HCV: Hepatitis C virus
HBV: Hepatitis B virus
HIV: Human immunodeficiency virus
CT: Computed tomography
FNAC: Fine-needle aspiration cytology.

## References

[1] *Rosai, Ackerman’s: Surgical Pathology Volume 1*. 10th edition. Elsevier Mosby; 2011:488–491.

[2] Hoyes AD, Kershaw DR (1985). Anatomy and development of the thyroid gland. Ear Nose Throat J.

[3] Mills SE (2007). Histology for Pathologists.

[4] Paludetti G, Galli J (1991). Tiroidi ectopiche. Acta Otorhinol Ital.

[5] Choudhury BK, Saikia UK (2011). Dual ectopic thyroid with normally located thyroid: a case report. J Thyroid Res.

[6] Eli SU, Marnane C (2011). Ectopic, submandibular thyroid causing hyperthyroidism. J Laryngol Otol.

[7] Mace ATM, Mclaughlin I, Gibson IW, Clark LJ (2003). Benign ectopic submandibular thyroid with a normotopic multinodular goitre. J Laryngol Otol.

[8] Ulug T, Ulubil SA, Alagol F (2003). Dual ectopic thyroid: report of a case. J Laryngol Otol.

[9] Toso A, Colombani F, Averono G, Aluffi P, Pia F (2009). Lingual thyroid causing dysphagia and dyspnoea. Case reports and review of the literature. ACTA otorhinolaryngologica italica.

[10] Léger J, Marinovic D, Garel C, Bonaïti-Pellié C, Polak M, Czernichow P (2002). Thyroid developmental anomaliesin first degree relatives of children with congenital hypothyroidism. J Clin Endocrinol Metab.

[11] Kousta E, Konstantinidis K, Michalakis C, Vorias M, Sambalis G, Georgiou M (2005). Ectopic thyroid tissue in the lower neck with a coexisting normally located multinodular goiter. Brief Lit Rev Hormones.

[12] Talwar N, Mohan S, Ravi B, Andley M, Kumar A (2008). Lithium-induced enlargement of a lingual thyroid. Singapore Med J.

[13] Di Benedetto V (1997). Ectopic thyroid gland in the submandibularregion simulating a thyroglossal duct cyst: a case report. J Pediatr Surg.

[14] Kumar R, Sharma S, Marwah A, Moorthy D, Dhanwal D, Malhotra A (2001). Ectopic goiter masquerading as submandibular gland swelling: a case report and review of the literature. Clin Nucl Med.

[15] Pollice L, Caruso G (1986). Struma cordis. Ectopic thyroid goiter inthe right ventricle. Arch Pathol Lab Med.

[16] Porqueddu M, Antona C, Polvani G, Pompilio G, Cavoretto D, Gianolli L, Arena V, Sala A, Biglioli P (1995). Ectopic thyroid tissue in the ventricularoutflow tract: embryologic implications. Cardiology.

[17] Ferlito A, Giarelli L, Silvestri F (1988). Intratracheal thyroid. J Laryngol Otol.

[18] Van VG (2003). Development of the thyroid gland: lessons from congenitally hypothyroid mice and men. Clin Genet.

[19] Anuj J, Sujata P (2010). Rare developmental abnormalities of thyroid gland, especially multiple ectopia: a review and our experience. Indian J Nucl Med.

[20] Sauk JJ (1970). Ectopic lingual thyroid. J Pathol.

[21] Mysorekar VV, Dandekar CP, Sreevathsa MR (2004). Ectopic thyroid tissue in the parotid salivary gland. Singapore Med J.

[22] Richards PS, Ahuja AT, King AD (2004). Clinics in diagnostic imaging (101): multinodular accessory thyroid tissue. Singapore Med J.

[23] Larochelle D, Arcand P, Belzile M, Gagnon NB (1979). Ectopic thyroid tissue-a review of the literature. J Otolaryngol.

[24] Kumar R, Gupta R, Bal CS, Khullar S, Malhotra A (2000). Thyrotoxicosis in a patient with submandibular thyroid. Thyroid.

[25] Aguirre A, de la Piedra M, Ruiz R, Portilla J (1991). Ectopic thyroid tissue in the submandibular region. Oral Surg Oral Med Oral Pathol.

[26] Steinwald OP, Muehrcke RC, Economou SG (1970). Surgical correction of complete lingual ectopia of the thyroid gland. Surg Clin North Am.

[27] Yoon JS, Won KC, Cho IH, Lee JT, Lee HW (2007). Clinical characteristics of ectopic thyroid in Korea. Thyroid.

[28] Gallo A, Leonetti F, Torri E, Manciocco V, Simonelli M, DeVincentiis M (2001). Ectopic lingual thyroid as unusual cause of severe dysphagia. Dysphagia.

[29] Hafidh MA, Sheahan P, Khan NA, Colreavy M, Timon C (2004). Role of CO2 laser in the management of obstructive ectopic lingual thyroids. J Laryngol Otol.

[30] Mussak EN, Kacker A (2007). Surgical and medical management of midline ectopic thyroid. Otolaryngol Head Neck Surg.

[31] Hari CK, Brown MJ, Thompson I (1999). Tall cell variant of papillary carcinoma arising from ectopic thyroid tissue in the trachea. J Laryngol Otol.

[32] Sand J, Pehkonen E, Mattila J, Seppănen S, Salmi J (1996). Pulsating mass at the sternum: a primary carcinoma of ectopic mediastinal thyroid. J Thorac Cardiovasc Surg.

[33] Jarvis J (1969). Lingual thyroid: a report of three cases and discussion. S Afr Med J.

[34] Abdallah-Matta MP, Dubarry PH, Pessey JJ, Caron P (2002). Lingual thyroid and hyperthyroidism: a new case and review of the literature. J Endocrinol Investig.

[35] Hazarika P, Siddiqui SA, Pujary K, Shah P, Nayak DR, Balakrishnan R (1998). Dual ectopic thyroid: a report of two cases. J Laryngol Otol.

[36] Batsakis JG, El-Naggar AK, Luna MA (1996). Thyroid gland ectopias. Ann Otol Rhinol Laryngol.

[37] Farrell ML, Forer M (1994). Lingual thyroid. Aust N Z J Surg.

[38] Thomas G, Hoilat R, Daniels JS, Kalagie W (2003). Ectopic lingual thyroid: a case report. Int J Oral Maxillofac Surg.

[39] Katz AD, Zager WJ (1971). The lingual thyroid: its diagnosis and treatment. Arch Surg.

[40] Conklin WT, Davis RM, Dabb RW, Reilly CM (1981). Hypothyroidism following removal of a “thyroglossal duct cyst”. Plast Reconstr Surg.

[41] Pritchyk KM, Thompson LD, Malekzadeh S (2004). Endoscopic managementof intratracheal ectopic thyroid. Otolaryngol Head Necksurg.

[42] Weiss SD, Orlich CC (1991). Primary papillary carcinoma of a thyroglossal duct cyst. Report of a case and review of the literature. Br J Surg.

[43] Katz AD, Hachigian M (1988). Thyroglossal duct cysts. A thirty-year experience with emphasis on occurrence in older patients. Am J Surg.

